# Prognostic value of microvolt T-wave alternans in a real-world ICD population. Twente ICD Cohort Studie (TICS)

**DOI:** 10.1007/s12471-014-0583-3

**Published:** 2014-08-14

**Authors:** K. Kraaier, M. A. G. M. Olimulder, P. F. H. M. van Dessel, A. A. M. Wilde, M. F. Scholten

**Affiliations:** 1 Department of Cardiology, Thoraxcentrum Twente, Medisch Spectrum Twente, Haaksbergerstraat 55, 7513 ER Enschede, the Netherlands; 2 Department of Cardiology, Academic Medical Center, Amsterdam, the Netherlands

**Keywords:** Microvolt T wave alternans, Implantable cardioverter defibrillator, Prognostic value, Mortality, Cardiomyopathy, Risk stratification

## Abstract

**Introduction:**

Survival benefit from ICD implantation is relatively low in primary prevention patients. Better patient selection is important to maintain maximum survival benefit while reducing the number of unnecessary implants. Microvolt T-wave alternans (MTWA) is a promising risk marker. In this study, we aimed to evaluate the predictive value of MTWA in ICD patients.

**Methods and results:**

This study was a substudy of the Twente ICD Cohort Study (TICS). Patients with ischaemic or non-ischaemic left ventricular dysfunction who received an ICD following current ESC guidelines were eligible for inclusion. Exercise-MTWA was performed and classified as non-negative or negative. The primary endpoint was the composite of mortality and appropriate shock therapy. Analysis was performed in 134 patients (81 % male, mean age 62 years, mean ejection fraction 26.5 %). MTWA was non-negative in 64 %. There was no relation between non-negative MTWA testing and mortality and/or appropriate shock therapy (all *p*-values >0.15). Due to clinical conditions, 24 % were ineligible for testing. These patients experienced the highest risk for mortality (*p* < 0.01).

**Conclusion:**

Non-negative MTWA testing did not predict mortality and/or appropriate shock therapy. Furthermore, MTWA testing is not feasible in a large percentage of patients. These ineligible patients experience the highest risk for mortality.

## Introduction

Several primary and secondary prevention trials have established the beneficial role of implantable cardioverter defibrillator (ICD) therapy in preventing sudden cardiac death (SCD) [1–6]. Therefore, the guidelines indicate ICD implantation in both survivors of cardiac arrest (secondary prevention) and in patients who are at high risk of developing life-threatening ventricular arrhythmias (primary prevention) [7].

Nevertheless, only a minority of patients will experience life-threatening ventricular arrhythmias (VA) necessitating ICD therapy, this being even more prominent in primary prevention patients [5, 6, 8, 9]. Improved patient selection is important to maintain maximum survival benefit of ICD implantation while reducing the number of unnecessary implants.

Microvolt T-wave alternans (MTWA) is a promising electrocardiographic risk marker for predicting SCD and life-threatening VA [10–14]. MTWA is a phenomenon of beat-to-beat variability in the amplitude of the T-wave. The precise underlying relation between MTWA and VA is not well known. One hypothesis suggests that MTWA reflects spatial and temporal heterogeneity or dispersion in the ventricular repolarisation, which could lead to VA by means of formation of functional re-entry circuits [15]. Despite conflicting results regarding its predictive value and feasibility [16–20], MTWA testing is currently incorporated in the guidelines as a diagnostic tool to improve risk stratification in patients with ischaemic and non-ischaemic severe left ventricular dysfunction [7, 21].

In this study, we aimed to prospectively evaluate the predictive value of MTWA in a real-life population of ICD recipients.

## Methods

### Study design and patient enrolment

The Twente ICD Cohort Study (TICS; NL13939.044.06) is a prospective single-centre observational study of ICD recipients, designed to evaluate the prognostic value of MTWA and other potential arrhythmic risk factors in predicting mortality and life-threatening VA. All consecutive patients between September 2007 and March 2010 who received an ICD for primary or secondary prevention of SCD according to the ESC guidelines were eligible for inclusion in the TICS [7]. In the currently presented MTWA substudy, only patients with ischaemic or non-ischaemic left ventricular dysfunction were included. Ischaemic heart disease was defined as left ventricular dysfunction associated with a documented history of myocardial infarction, prior coronary artery bypass surgery, prior percutaneous coronary intervention or significant narrowing of at least one of the major coronary arteries. All other cases were classified as non-ischaemic left ventricular dysfunction. The choice and programming of the device were left to the discretion of the implanting physician. Standard settings for our centre are ventricular fibrillation (VF) zone >230 beats per minute (bpm), and ventricular tachycardia (VT) zone >185 bpm with antitachycardia pacing (ATP).

### T-wave alternans testing

MTWA testing was performed in all patients in sinus rhythm. Chronic medication, including beta-blockers, was continued during MTWA testing. Careful skin preparation was performed and high-resolution electrodes were used to minimise noise. In addition to the standard 12-lead ECG, three orthogonal X, Y and Z leads were recorded as well. Measurements were made with a HeartTWave system II (Cambridge Heart Inc., Bedford, Massachusetts, USA) using the spectral analysis method during exercise. After gradually increasing the workload to achieve a constant heart rate, a target heart rate between 100 and 110 bpm was attained and kept stable for 2.5 min. Subsequently, during 1.5 min, a target heart rate between 110 and 120 bpm was maintained. The result of the MTWA test was automatically interpreted by the Alternans Report Classifier within the system and carefully reviewed by trained physicians. Each MTWA report was classified positive, indeterminate or negative using accepted criteria [22, 23]. A test was defined positive if the MTWA voltage was ≥1.9 μV for at least 1 min with an onset heart rate <110 bpm or at rest in any of three orthogonal leads (X, Y or Z), or in two adjacent precordial leads. If the recording did not prove positive and the heart rate was >105 bpm for at least one minute, the MTWA test was defined as negative. An MTWA test was considered indeterminate if the test did not meet the criteria for being classified as positive or negative. Based on prior literature about further analysis, patients with indeterminate or positive tests were combined as non-negative MTWA [24].

### Follow-up and endpoints

All patients were followed on a regular basis (every 3–6 months) in our outpatient ICD clinic, either by visits or telemonitoring. All ICD data were obtained by interrogation of the device.

The primary endpoint was the combined endpoint of all-cause mortality and appropriate ICD shock therapy for VT or VF. Secondary endpoints were all-cause mortality and appropriate ICD shock therapy for VT or VF.

### Statistical analysis

Continuous variables are presented as mean ± SD, and categorical data are summarised as frequencies and percentages. Differences in baseline characteristics between MTWA positive and negative patients were analysed using Student’s t-test or the Mann–Whitney U-test, as appropriate, if continuous, or chi-square or Fisher’s exact test if categorical. Unless otherwise specified, p-values and confidence intervals (CIs) were two-sided and a *p*-value <0.05 was considered significant.

Univariate and multivariate logistic regression analyses were performed to evaluate MTWA as an independent predictor of mortality and/or appropriate shock. All variables were evaluated by univariate analysis as possible predictors, and only those with a significance at or below *p* = 0.15 were analysed using multivariate logistic regression analysis.

## Results

### Patients

From September 2007 until March 2010, 503 patients received an ICD. Of these patients, 300 were included in the TICS. In all the other patients, no informed consent was obtained. Of these 300 patients, 269 patients with ischaemic or non-ischaemic left ventricular dysfunction were eligible for this MTWA substudy. MTWA testing was performed in 134 of 269 (49.6 %) patients. In 62 of 269 (23.0 %) patients MTWA was not performed due to logistic reasons (mainly secondary prevention ICD recipients who received their ICD during hospitalisation), 7 of 269 (2.6 %) patients refused to participate and 66 of 269 (24.5 %) patients were not capable of performing exercise-based MTWA testing.

In the 134 tested patients (81 % male, mean age 62 years, mean left ventricular ejection fraction (LVEF) 26.5 %), MTWA was positive in 48 (35.8 %) patients, negative in 48 (35.8 %) patients and indeterminate in 38 (28.3 %) patients (due to noise (9 (6.7 %) patients), frequent ventricular or atrial ectopy (15 (11.2 %) patients), or not achieving the target heart rate (14 (10.4 %) patients). Positive and indeterminate results were combined in the non-negative group. The clinical characteristics are presented in Table 1. Among patients with a non-negative test result, significantly more were male and had wider QRS duration. There was a trend for association between lower LVEF and non-negative MTWA.

**Table 1:** Clinical characteristics

	All	Negative	Non-negative	Ineligible
Number of patients	200	48	86	66
General				
Age (years ± SD)	63.0 (10.3)	59.8 (11.9)	63.0 (9.5)	65.8 (9.8)**
Gender (male)	170 (84.6)	33 (68.8)	75 (87.2)*	62 (93.9)**
LVEF (% ± SD)	26.7 (12.2)	29.1 (13.1)	25.0 (11.2)	27.0 (12.8)
Comorbidities				
Hypertension	53 (26.4)	12 (25.0)	24 (27.9)	18 (27.3)
DM	47 (23.4)	11 (22.9)	14 (16.3)	22 (33.3)
COPD	21 (10.4)	2 (4.2)	11 (12.8)	7 (10.6)
CVA/TIA	14 (7.0)	3 (6.2)	4 (4.7)	7 (10.6)
AF	52 (25.9)	5 (10.4)	9 (10.5)	40 (60.6)**
Indication				
Primary	161 (80.5)	38 (79.2)	74 (86.0)	49 (74.2)
Secondary	39 (19.5)	10 (20.8)	12 (14.0)	17 (25.8)
Aetiology				
Ischaemic	135 (67.2)	34 (70.8)	53 (61.6)	48 (74.2)
Dilated	66 (32.8)	14 (29.2)	44 (38.4)	17 (25.8)
Medication				
Beta-blocker	169 (84.1)	42 (87.5)	70 (81.4)	55 (83.3)
ACEi/ARB	169 (84.1)	50 (83.3)	75 (87.2)	53 (80.3)
Diuretics	153 (76.1)	32 (66.7)	66 (76.7)	55 (83.3)*
Amiodarone	20 (10.0)	4 (8.3)	6 (7.0)	10 (15.2)
NYHA functional class ≥3	53 (26.8)	7 (14.6)	18 (21.2)	29 (45.3)**
QRS duration (ms ± SD)	125 (31)	115 (30)	125 (28)	131 (34)*

*p < 0.05 and **p < 0.01 in comparison with TWA negative group

*LVEF* left ventricular ejection fraction, *DM* diabetes mellitus, *COPD* chronic obstructive pulmonary disease, *CVA* cerebrovascular accident, *TIA* transient ischaemic attack, *AF* atrial fibrillation, *ACEi* angiotensin-converting enzyme inhibitor, *ARB* angiotensin receptor blocker, *NYHA* New York Heart Association

### Clinical outcome

During a mean follow-up of 38 ± 10 months, 21 patients (15.6 %) reached the primary endpoint of death (10 (7.5 %) patients) and/or appropriate shock therapy (11 (8.2 %) patients).

**Fig. 1:**
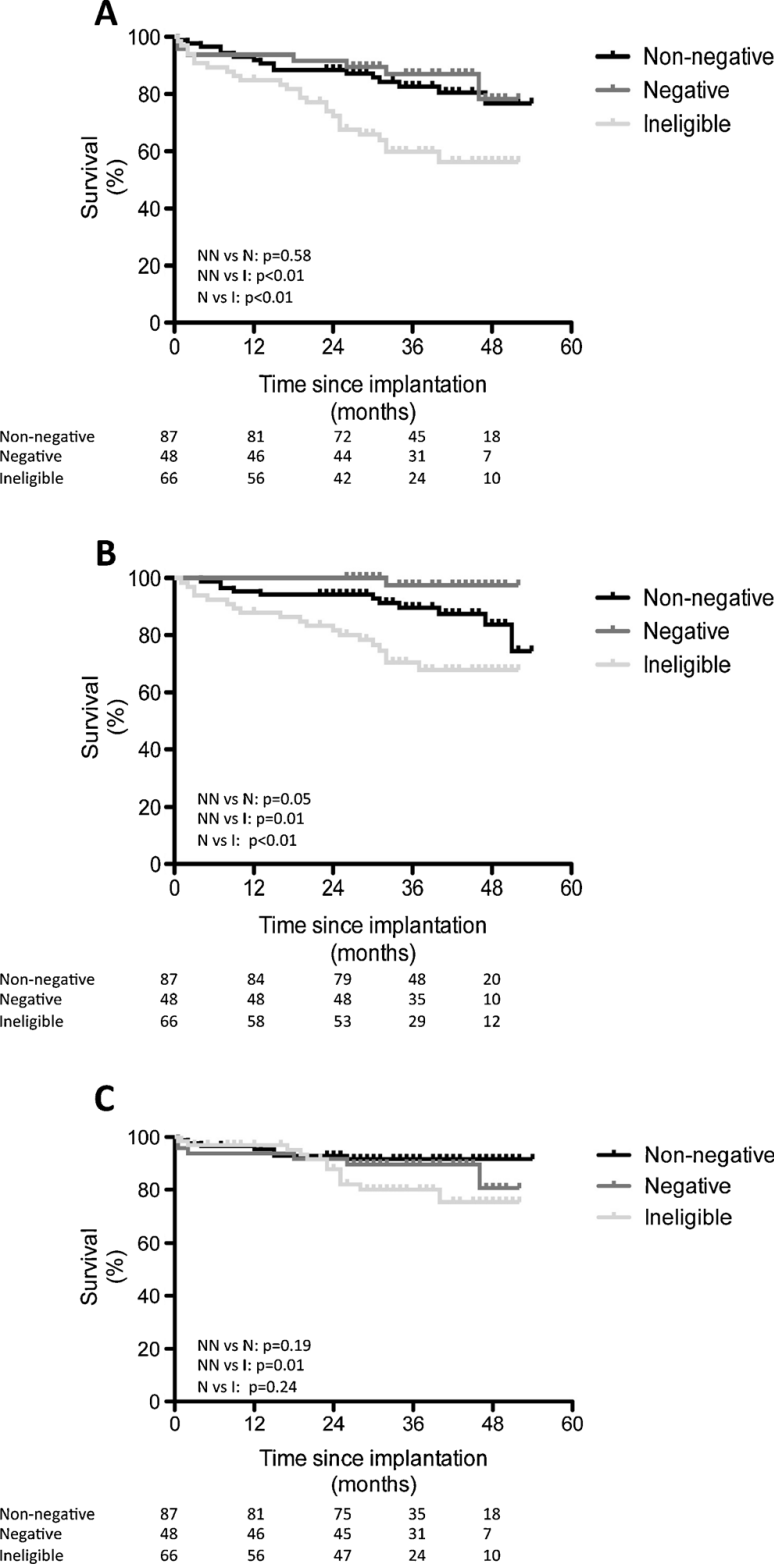
Survival curves regarding the role of MTWA in predicting **a** mortality and/or appropriate shock therapy, **b** mortality and **c** appropriate shock therapy

Predictors for the combined endpoint of mortality and appropriate shocks (Table 2) were wider QRS duration (*p* = 0.03) and chronic obstructive pulmonary disease (*p* = 0.01). No significant relation was found between non-negative MTWA testing and the primary endpoint (*p* = 0.58, Fig. 1).

**Table 2:** Predictors for the combined endpoint of mortality and appropriate therapy

	Univariate analysis	
	Hazard ratio	*p*
Age	1.03 (0.99–1.07)	0.16
Male gender	1.13 (0.42–3.04)	0.89
LVEF	0.99 (0.96–1.03)	0.67
Comorbidities		
Hypertension	0.54 (0.18–1.59)	0.26
DM	1.25 (0.46–0.37)	0.66
COPD	3.47 (1.36–8.84)	0.01
CVA/TIA	0.04 (0.00–64.08)	0.40
AF	1.51 (0.45–5.10)	0.50
Prophylactic indication	0.55 (0.22–1.39)	0.21
Ischaemic cardiomyopathy	0.74 (0.33–1.69)	0.47
NYHA functional class ≥3	1.22 (0.45–3.30)	0.69
QRS duration (ms ± SD)	1.01 (1.00–1.03)	0.03
Non-negative MTWA	1.29 (0.53–3.13)	0.58

Hazard ratio (±95 % CI). Hazard ratio for age per 1 year increase in age. Hazard ratio for LVEF per 1 % increase in LVEF. Hazard ratio for QRS duration per 1 ms increase in duration

*LVEF* left ventricular ejection fraction, *DM* diabetes mellitus, *COPD* chronic obstructive pulmonary disease, *CVA* cerebrovascular accident, *TIA* transient ischaemic attack, *AF* atrial fibrillation, *ACEi* angiotensin-converting enzyme inhibitor, *ARB* angiotensin receptor blocker, *NYHA* New York Heart Association

Ischaemic left ventricular dysfunction was present in 87 (64.9 %) patients, of whom 53 (60.9 %) had a non-negative test result. Non-ischaemic left ventricular dysfunction was present in 47 (35.1 %) patients, of whom 33 (70.2 %) had a non-negative test result. MTWA was neither predictive for the combined endpoint of death and/or appropriate shock in ischaemic or non-ischaemic LV dysfunction (all *p*-values >0.66).

Regarding the secondary endpoint mortality, significant associations were found with lower LVEF (*p* = 0.03), chronic obstructive pulmonary disease (*p* = 0.05), NYHA functional class ≥ 3 (*p* = 0.02), non-negative MTWA (*p* = 0.05, Fig. 1) or QRS duration (*p* = 0.03). After constructing a multivariate model using these parameters, multivariate analysis showed that MTWA was no longer an independent predictor (*p* = 0.15). In the patients with ischaemic left ventricular dysfunction, 6 (6.9 %) patients died, all in the non-negative MTWA group. Using Kaplan-Meier survival analysis, a significant relation between non-negative MTWA and mortality was found (*p* = 0.04). Other significant univariate variables were lower LVEF and wider QRS duration. After correction using multivariate regression analysis, non-negative MTWA lost its significance. In the non-ischaemic LV dysfunction group, 5 (10.6 %) patients died, four in the non-negative group and one in the negative group. No relation was found between MTWA and mortality (*p* = 0.55).

Appropriate shock therapy as alternative secondary endpoint occurred in 11 (8.2 %) patients, 5 (5.8 %) patients with a non-negative MTWA and 6 (12.5 %) patients with a negative MTWA (*p* = 0.19, Fig. 1). In the patients with ischaemic left ventricular dysfunction, 8 (9.2 %) patients experienced appropriate shock therapy. Three (37.5 %) patients had a non-negative test, and 5 (62.5 %) had a negative test (*p* = 0.14). In the patients with non-ischaemic LV dysfunction, 3 (6.3 %) patients experienced appropriate shock therapy. Two events (66 %) occurred in patients with a non-negative test result whereas 1 (33 %) patient with a negative test result experienced appropriate therapy (ns, *p* = 0.97).

### Ineligible patients

In 66 of 269 (24.5 %) patients, MTWA by means of exercise stress testing was technically not feasible because of atrial fibrillation (*n* = 43), pacemaker-dependency (*n* = 8), or clinical state (*n* = 15). Ineligible patients were more frequently male (*p* = 0.01), older (*p* < 0.01), more often known with atrial fibrillation (*p* < 0.001), and a lower functional class (*p* < 0.001). The combined primary endpoint of mortality and appropriate shock was reached in 26 (39.4 %) patients. This was significantly higher than in the non-negative (RR 2.5 (95 % CI 1.3-4.7) *p* < 0.01) and negative (RR 3.3 (95 % CI 1.4-7.6), *p* < 0.01) MTWA group. After multivariate correction for confounders, ineligibility for TWA testing remained the only significant predictor of the combined endpoint of mortality and appropriate shock therapy (Fig. 1).

Regarding the secondary endpoints, compared with non-negative patients, ineligible patients experienced both higher rates of mortality (*p* = 0.01, Fig. 1) and appropriate shocks (*p* = 0.01, Fig. 1).

## Discussion

In this study, non-negative MTWA was not associated with mortality and/or appropriate shock therapy. In fact, there was no relation between VA requiring ICD shock therapy and non-negative MTWA. Furthermore, patients—in whom MTWA is not technically feasible—experienced highest risk for mortality and appropriate shock therapy.

The role of MTWA testing in selecting patients for ICD implantation remains controversial. Some studies state that MTWA is a potentially useful predictor of VA and mortality in patients with ischaemic or non-ischaemic heart disease [10–14]. Other studies reported results which are less promising. The relation with mortality was still significant, but no relation between MTWA and VA was found [16]. The results of the MTWA substudy of the SCD-HeFT are in concordance with our study, namely not showing a relation between MTWA and mortality or appropriate ICD therapy [17]. The differences in outcome with prior studies which did find significant relations between MTWA and SCD [10–14] can possibly be explained by the use of appropriate ICD therapy as a surrogate endpoint for arrhythmic mortality which is an overestimation, and the use of beta-blockers during exercise testing although this is recommended in the current consensus [16, 17, 21].

Recently, doubt has been raised about the feasibility of MTWA testing in potential ICD recipients [18]. In our study, MTWA testing was not technically feasible in 24.5 % of included patients. In the literature, percentages between 35 and 49 % can be found [18–20]. If testing in ineligible patients is mandatory, MTWA can be assessed using a protocol based on pacing or pharmacological increase of heart rate. These protocols, however, are not useable in patients with atrial fibrillation or other irregular heart rhythms, but are very useful for patients who cannot reach the target heart rate. Concordance between these different protocols varies between 55 and 98 % [25–29]. Not much is known about the prognostic value of pacing-based MTWA. Rashba et al. found no relation with death, sustained ventricular arrhythmia or appropriate ICD therapy using an atrial paced protocol [26]. No data are reported on the prognostic value of ventricular pacing-based protocols or pharmacological intervention protocols to increase heart rate.

The question remains whether these patients need to be tested by means other than exercise stress testing MTWA protocols since in both our study and the studies by Shizuta and Jackson, patients in whom exercise-based MTWA testing is not possible experience the highest risk of mortality and VA requiring ICD therapy, which remains statistically significant after correction for possible confounders [19, 20].

### Limitations

The main limitation of this trial is the number of patients in combination with the low event rate. The study is therefore relatively underpowered and caution is required when interpreting the results.

## Conclusion

In the present study, we did not find a significant relation between non-negative MTWA testing and mortality or appropriate shock therapy. Its role in the selection of ICD patients therefore remains controversial. Furthermore, MTWA testing is not feasible in a large percentage of patients. These ineligible patients experience the highest risk for mortality or appropriate ICD therapy.
